# Treatment of Parkinson's disease by deep brain stimulation: a bibliometric analysis

**DOI:** 10.1590/1516-3180.2023.0187.R1.04032024

**Published:** 2024-05-31

**Authors:** Denise Maria Meneses Cury Portela, Ana Raquel Batista de Carvalho, Antonio Rosa de Sousa, Clarice Listik, Daniela Reis Joaquim de Freitas, Maria Eliete Batista Moura, Gustavo Sousa Noleto

**Affiliations:** I MSc. Physician, Professor, Medicine Course, Department of Health, Centro Universitário Uninovafapi, Teresina, Piauí, Brazil.; II MSc. Doctoral student, Post-graduate Nursing program, Universidade Federal do Piauí (UFPI), Teresina, Piauí, Brazil.; III Nurse. Master's student, Post-graduate Nursing program, Universidade Federal do Piauí (UFPI), Teresina, Piauí, Brazil.; IV MSc. Physician, Doctoral Student, Center for Movement Disorders, Department of Neurology, Universidade de São Paulo (USP), São Paulo, Brazil.; V PhD. Professor, Department of parasitology and microbiology, Universidade Federal do Piauí (UFPI), Teresina, Piauí, Brazil.; VI PhD. Nurse, Professor, Post-graduate Nursing Program, Universidade Federal do Piauí (UFPI), Teresina, Piauí, Brazil.; VII PhD. Physician, Department of Neurosurgery, Medical School, Universidade de São Paulo (USP), São Paulo, Brazil.

**Keywords:** Deep Brain Stimulation, Parkinson's disease, Bibliometrics, Parkinsonism, Primary, Paralysis Agitans, Bibliometric Indicators

## Abstract

**BACKGROUND:**

For more than 30 years, deep brain stimulation (DBS) has been a therapeutic tool for Parkinson's disease (PD) treatment. DBS can ameliorate several motor and non-motor symptoms and improve the patients’ quality of life.

**OBJECTIVES:**

To analyze the global scientific production of original and review articles on Parkinson's disease treatment using deep brain stimulation.

**DESIGN AND SETTING:**

Descriptive, bibliometric study with a quantitative approach.

**METHOD:**

The research protocol was conducted in March 2023 using the Web of Science database. Six hundred eighty-four articles were included in the analysis. Data were imported into RStudio Desktop Software, linked to R Software. The Bibliometrix R package, its Biblioshiny web interface, and VOSviewer software were used for the analysis.

**RESULTS:**

The international production began in 1998. Movement Disorders is the journal with the largest number of published articles and the most cited. Michael Okun and Andres Lozano are the authors who produced the most in this area. The University of Florida is the most active affiliated institution in Brazil. The United States has the largest number of collaborations and is mainly published by local researchers. In contrast, countries such as the United Kingdom and Canada have a high number of multi-country publications. The 15 most cited studies predominantly investigated subthalamic nucleus stimulation.

**CONCLUSION:**

DBS for Parkinson's disease is a relatively novel therapeutic approach, with studies that have expanded over the last twenty-five years. Most scientific production was quantitative and restricted to specialized journals. The United States, Europe, and China held the most articles.

## INTRODUCTION

Parkinson's disease (PD) is a chronic neurological disorder that affects motor function and causes tremors, rigidity, and bradykinesia.^1^ Deep brain stimulation (DBS) is a surgical treatment for PD, especially for patients or those who experience significant side effects. It improves both motor and non-motor symptoms as well as the patient's quality of life.^2^

Research on the use of DBS in the treatment of PD has evolved exponentially over the years. In the 1990s, stimulation of the subthalamic nucleus (STN) was effective in reducing motor symptoms in patients with advanced disease.^3,4^ Studies have focused on establishing the best parameters for DBS therapy and analyzing its long-term effects.^5,6^

Bibliometric studies are used to assess the impact of research topics, identify research trends, evaluate researchers and institutions, and inform research policies. Thus, bibliometric studies provide a way to objectively measure the impact and productivity of research and can help inform decision-making regarding research policies and funding.

## OBJECTIVE

To analyze the worldwide scientific production of original and review articles on Parkinson's disease treatment by deep brain stimulation.

## METHODS

### Research design

This article is a descriptive bibliometric study with a quantitative approach guided by the five recommended steps in bibliometric research.^7^ Since this is a bibliometric study, no ethics committee approval was required.

The use of bibliometric analyses has been growing in the field of health, particularly in neurology. The literature points to bibliometric analyses on various topics, such as movement disorders,^8^ dystonia,^9^ Parkinson's disease stem cells,^10^ and deep brain stimulation.^11^ Notably, this type of analysis allows the investigation of more data than systematic literature reviews, while maintaining high rigor, scientific soundness, transparency, and replicability.^12,13^

### Data-gathering period

Scientific articles were searched using an advanced query in the Web of Science™ (WoS) database on March 19, 2023. WoS was used in this study because of its international academic recognition. It is considered one of the most comprehensive scientific bases, that pioneered the aggregation of journals from more than 100 areas of knowledge.^14^

### Selection criteria

Original and review articles published before March 19, 2023, were included in the study. In addition, documents that deviated from the scope of the research, opinion articles, reflection articles, editorials, and case studies were excluded.

### Data-gathering

To formulate the search strategy, both controlled and uncontrolled descriptors from Medical Subject Headings (MeSH) were used, along with search operators and wildcard characters. In WoS, the search included only the titles of the documents, following the approach used in other studies, to increase the search accuracy of the search and reduce false-positive results.^15,12^

**Figure 1:**
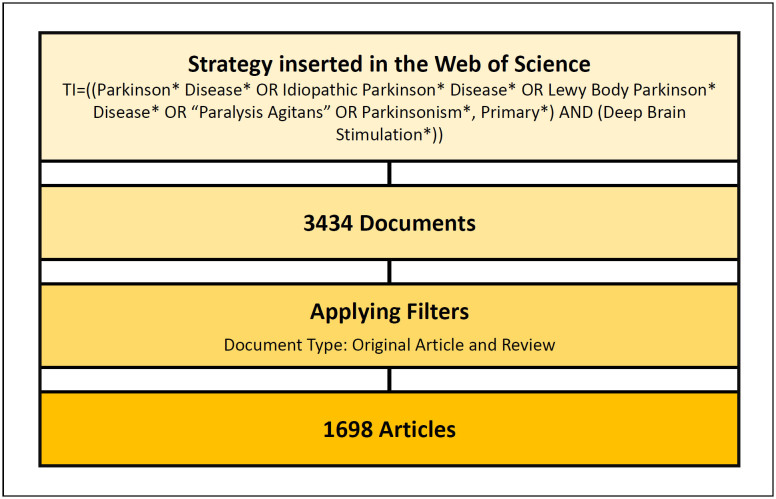
Search strategy used and selection of articles included.

Thus, the search first resulted in 3434 articles, after filtering and applying the previously described criteria, 1,698 articles remained, which had all available information downloaded in text file format for analysis. Figure 1 shows both the search strategy and the selection process of the included articles.

### Data processing and analysis

The recovered data were imported into RStudio Desktop Software, version 2023.03.0+368 (©Posit Software, Massachusetts, United States, 2023), linked to R Software, version 4.2.3 (The R Foundation, Vienna, Austria, 2023). For the analysis, we used the Bibliometrix R package (© K-Synth Srl, Academic Spin-Off of the University of Naples Federico II, Naples, Italy, 2023) and the Biblioshiny application, which provides a web interface for Bibliometrix.^16^

In this study, we sought to elucidate on: the number of articles published per year, the scientific journals that published the most and those that were most cited, the productivity of authors according to Lotka's Law,^17^ and according to time, the most productive institutions and countries, collaboration rate, the most cited articles, the conceptual structure, and thematic evolution using KeyWords Plus™.

## RESULTS

As described previously, 1,698 articles were analyzed in the present study. The search was carried out for the period of 1945 to 2023 and identified the first result of an article published in 1998; for this reason, the period considered was from 1998 to 2023.

**Figure 2:**
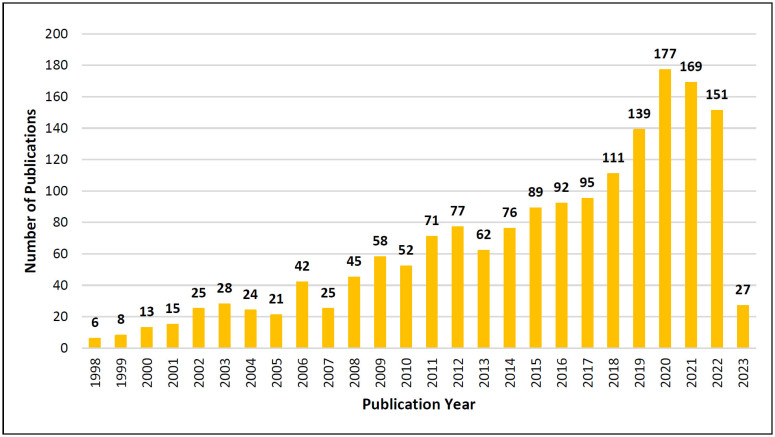
Annual distribution of articles according to year of publication.

The evolution of scientific production on PD and DBS is shown in Figure 2, which graphically displays the annual number of publications in the period studied, demonstrating that international interest in the subject has been constantly growing, reaching its peak in 2020 and despite a reduction in 2021, remains high.

**Figure 3:**
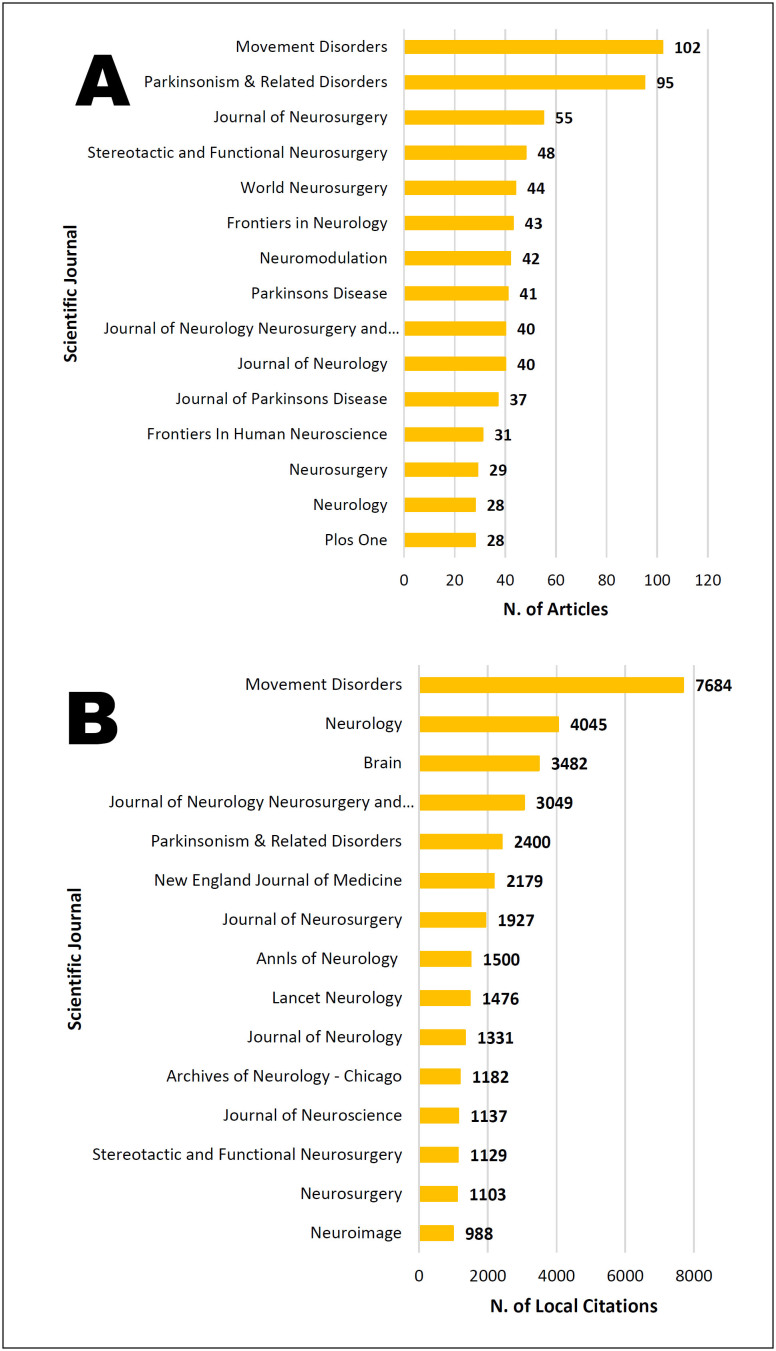
Scientific journals that published the most articles and most cited scientific journals.

When analyzing the articles, 358 scientific journals were identified. Figure 3A shows a list of the most representative journals regarding the number of publications. In contrast, Figure 3B shows the most cited journals in the references of the evaluated articles. The scientific journal Movement Disorders stood out for publishing the most articles on the subject and being the most cited.

**Figure 4:**
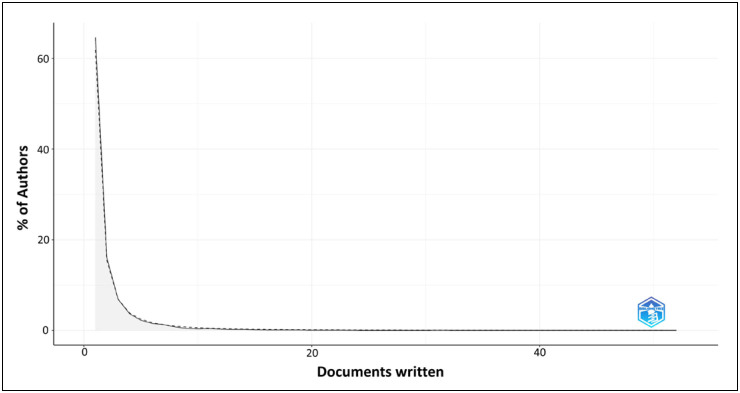
Productivity of scientists according to Lotka's Law.

Lotka's Law (Figure 4) describes the publication frequency of the authors on a given topic. According to this law, a large proportion of scientific literature is produced by a small number of authors and the production of many small producers is equal to a small number of large producers. In this study, the articles evaluated were produced by 5,923 authors. Among them, 65% published only one article, 32% published two to eight articles, and 3% published 9 to 52 articles, demonstrating compliance with Lotka's law.

**Figure 5:**
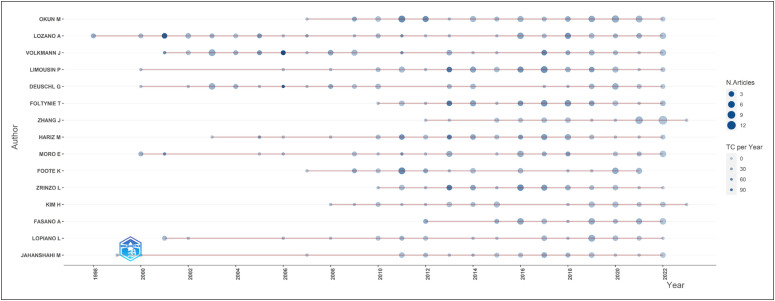
Top - Authors’ production over time.

Figure 5 shows the production (article publications) of the 15 principal authors over time. In this figure, the size of the bubble is proportional to the number of articles (larger bubbles indicate a more significant number of articles) and the intensity of the color is proportional to the total citations (TC) per year (dark blue indicates a more significant number of citations). In terms of the number of articles, Michael Okun (n = 52) was the most productive author, followed by Andres Lozano (n = 50), Jens Volkmann (n = 45), Patricia Limousin (n = 43), and Guenther Deuschl (n = 38). Consequently, the most cited were Andres Lozano (n = 98), Jens Volkmann (n = 112), and Guenther Deuschl (n = 99), with more than 90 citations.

**Figure 6:**
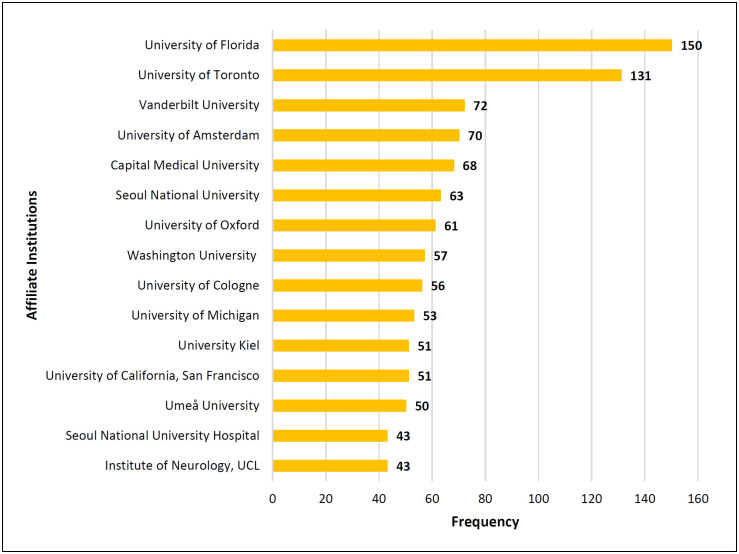
Institutions most involved in research on the subject.

When evaluating the authors’ affiliations, 1,863 institutions were identified. Therefore, Figure 6 presents the institutions most involved in research on the subject according to the co-occurrence of these institutions at the authors’ addresses. Notably, the same institution can be present more than once in each article. The results showed that 1,021 (54.8%) institutions appeared only once. Authors from the University of Florida were the most active on this research topic, appearing 150 times.

**Figure 7:**
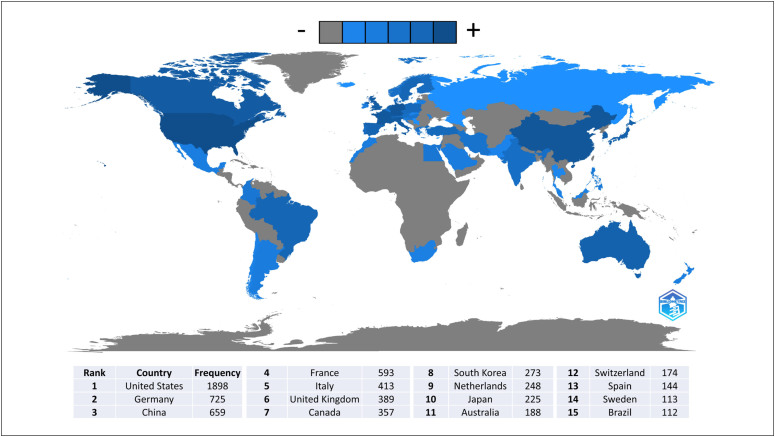
Scientific production by country according to authors’ affiliation.

Researchers who published on the subject were from 55 countries. The geographic distribution of the articles is shown in Figure 7. The value obtained was based on the co-occurrence of countries according to authors’ affiliations, which explains why the frequency was greater than the number of articles evaluated. In the figure, shades of blue, from lightest to darkest, indicate an increase in local authors, while gray indicates the absence of local authors.

When evaluating the collaboration rate (the ratio between the number of multi-country collaborations and the total number of articles attributed) based on the affiliation of the corresponding author (Table 1), it is evident that despite having carried out the highest number of collaborations, the United States mainly published with local researchers. In contrast, countries such as the United Kingdom and Canada have high rates of multi-country publications (51% and 50%, respectively).

**Table 1:** Production and collaborations of countries according to the corresponding author

Rank	Country	Articles	SCP	MCP	MCP_Rate
1	United States	435	353	82	19%
2	China	192	162	30	16%
3	Germany	170	123	47	28%
4	Italy	99	80	19	19%
5	France	92	66	26	28%
6	United Kingdom	92	45	47	51%
7	Canada	70	35	35	50%
8	Netherlands	55	38	17	31%
9	Japan	53	50	3	6%
10	Korea	51	45	6	12%
11	Spain	39	34	5	13%
12	Switzerland	35	23	12	34%
13	Australia	34	26	8	24%
14	Sweden	30	16	14	47%
15	Brazil	25	16	9	36%

Country = Country of the corresponding author's affiliation; Articles = Number of articles per country of corresponding author's affiliation; SCP = Single Country Publication; MCP = Multi-Country Publication; MCP_rate = Multi-Country Publication rate.

A total of 1,698 articles were cited 18,591 times, averaging 31.42 citations per item. The 15 most-cited articles ranged from 1,885 to 437 (Table 2). These articles were published in eight different journals between the years 2001 and 2010.

**Table 2:** Ranking of the most cited published articles on the subject.

Rank	Author (year), Journal	Title	Total Citations (TC)
1	Deuschl G et al. (2006), N Engl J Med^18^	A randomized trial of deep-brain stimulation for Parkinson's disease	1885
2	Obeso JA et al. (2001), N Engl J Med^19^	Deep-brain stimulation of the subthalamic nucleus or the pars interna of the globus pallidus in Parkinson's disease.	1161
3	Weaver FM et al. (2009), JAMA-J Am Med Assoc^20^	Bilateral deep brain stimulation vs. best medical therapy for patients with advanced Parkinson's disease: a randomized controlled trial	1025
4	Benabid AL et al. (2009), Lancet Neurol^21^	Deep brain stimulation of the subthalamic nucleus for the treatment of Parkinson's disease	897
5	Follett KA et al. (2010), N Engl J Med^22^	Pallidal versus subthalamic deep-brain stimulation for Parkinson's disease	862
6	Rodriguez-Oroz Mc et al. (2005), Brain^23^	Bilateral deep brain stimulation in Parkinson's disease: a multicentre study with 4 years follow-up	761
7	Little S et al. (2013), Ann Neurol^24^	Adaptive deep brain stimulation in advanced Parkinson's disease	715
8	Bronstein JM et al. (2011), Arch Neurol-Chicago^25^	Deep brain stimulation for Parkinson's disease: an expert consensus and review of key issues	575
9	Stefani A et al (2007), Brain^26^	Bilateral deep brain stimulation of the pedunculopontine and subthalamic nuclei in severe Parkinson's disease	541
10	Kumar R et al. (1998), Neurology^27^	Double-blind evaluation of subthalamic nucleus deep brain stimulation in advanced Parkinson's disease	500
11	Williams A et al. (2010), Lancet Neurol^28^	Deep brain stimulation plus best medical therapy versus best medical therapy alone for advanced Parkinson's disease (PD SURG trial): a randomized, open-label trial	493
12	Benabid AL et al. (2003), Curr Opin Neurobiol^29^	Deep brain stimulation for Parkinson's disease	486
13	Witt K et al (2008), Lancet Neurol^30^	Neuropsychological and psychiatric changes after deep brain stimulation for Parkinson's disease: a randomized, multicentre study	445
14	Odekerken VJJ et al. (2013), Lancet Neurol^31^	Subthalamic nucleus versus globus pallidus bilateral deep brain stimulation for advanced Parkinson's disease (NSTAPS study): a randomized controlled trial	437
15	Okun MS et al. (2009), Ann Neurol^32^	Cognition and mood in Parkinson's disease in subthalamic nucleus versus globus pallidus interna deep brain stimulation: the COMPARE trial	372

**Figure 8:**
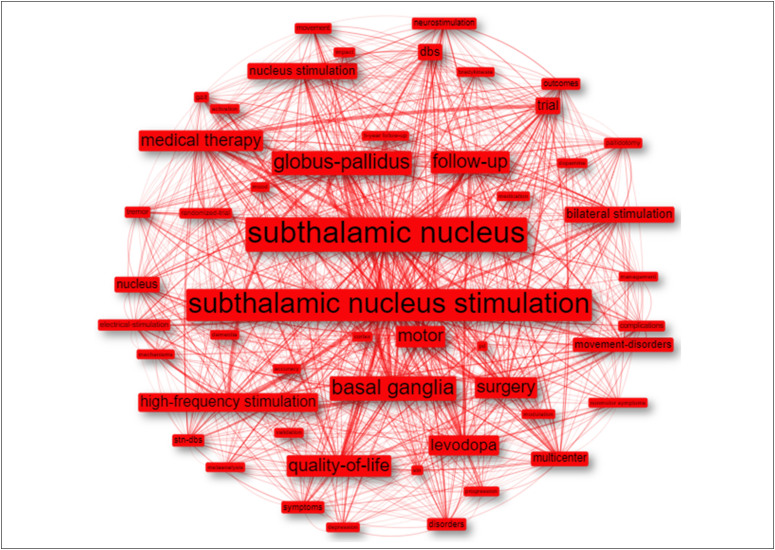
The conceptual structure according to KeyWords Plus™.

In bibliometric research, keywords can summarize the focus of articles and determine which subjects are being addressed, that is, their conceptual structures.^33^ Thus, to demonstrate the conceptual structure of the articles, the 50 most frequent KeyWords Plus™ were used to create Figure 8 (using the Leiden clustering algorithm), where the size of each box is proportional to the frequency of the term (the more a term appears, the larger its size will be).^34^ In the figure, the formation of just one cluster can be observed, demonstrating the centrality of the topic, with terms that refer to structures targeted in DBS surgery for treating PD, especially the subthalamic nucleus.

**Figure 9:**
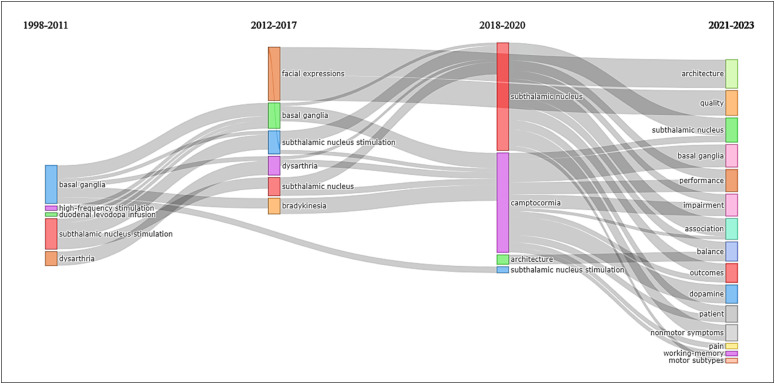
Thematic evolution according to KeyWords Plus™.

When observing the evolution of the theme (Figure 9), using the most frequent Keywords Plus™ again, it is evident that the first publications were more restricted (1998-2011), encompassing terms such as subthalamic nucleus (STN) stimulation, basal ganglia, dysarthria, high frequency, and duodenal infusion of levodopa. However, recently (2021-2023), there has been diversification in studies on PD and DBS, addressing targets, outcomes, quality of life, non-motor symptoms, and balance.

## DISCUSSION

This bibliometric analysis included articles that addressed the treatment of PD using deep brain stimulation for almost three decades (1998 2023). Notably, from 1990 to 2019, the number of patients diagnosed with PD increased from 2.5 million to 6.1 million globally, owing to population aging and environmental factors, which may have favored this growing incidence.^35^

The growing trend in annual publications indicates continued investment in DBS research for the treatment of PD. However, after 2020, there has been a decline in the number of publications despite growing scientific interest. The explanation for this decrease in studies may be related to the COVID-19 pandemic, which may have delayed the progress of research protocols because research related to the subject requires complex methodological designs that take time to execute and publish.

Regarding scientific journals with the most publications and citations, it was noted that they were mainly dedicated to neuroscience. Only one of the 15 most-cited journals (New England Journal of Medicine) was identified as a general medical journal. In addition, among the 15 journals that published the most articles, seven were also among the most cited. These findings indicate the predominance of journals in the subspecialty mentioned above, which excel in terms of the quantity and quality of published articles.

Although many authors have participated in producing evidence, most have published only one article (65%) and are considered occasional researchers in the area. Therefore, according to Lotka's Law, this theme must be consolidated. This law estimates that only 60% of authors produce a single document in consolidated areas, with an average of 3.5 documents per author. In the present study, 1,146 authors published 3.52 articles.^16^

Notably, the most productive author, Michael Okun, appeared twice in the citation ranking,^25,32^ with the most cited article of his career owning 575 citations and ranking eighth among the most cited.^25^ The second most productive author, Andrés Lozano, has five articles among the most cited, and his most cited article has 1161 citations.^23,25,27^

Most of these authors began publishing extensively after 2010. Lozano has the longest timeline, spanning from 1998 to 2023, and has been active in the field for almost three decades. Simultaneously, scholars such as Elena Moro, J Volkmann, Leonardo Lopiano, Marjan Jahanshahi, Limousin, and Deuschl, maintain their scientific activity in the area for long periods. Other authors, such as Jianguo Zhang and Alfonso Fasano, who are currently among the main researchers in the area, began publishing their research after 2010.

The University of Florida and the University of Toronto have prominent positions among the institutions that have conducted the most research on the subject, contributing consistent and systematic articles on the topic. It is essential to highlight that Michael Okun, from the University of Florida, and Andrés Lozano, from the University of Toronto, have already published together,^25^ demonstrating the importance of teamwork with different research centers of excellence, which have been publishing consistently for decades.

Although authors from different countries contributed, most of them were from the United States, China, and Europe (Germany, France, Italy, and the United Kingdom). However, the fact that the United States has the highest number of authors and a low collaboration rate is noteworthy, suggesting that its protocols are predominantly conducted between local institutions and are not shared with other countries.

Advanced PD has become the most studied and common indication for DBS, using different targets. The 15 most cited articles were related to STN stimulation. Of these, seven compared the STN to the Globus Pallidus Internus (GPi),^20-23,28,31,32^ which confirmed that these are the two most common targets for DBS and are both components of the basal ganglia-thalamo-cortical loop. Furthermore, stimulation of these sites has been associated with significant improvements in the cardinal motor signs of PD, including tremors, bradykinesia, and rigidity.^37^

The pedunculopontine nucleus (PPN) has also been evaluated and studied to treat axial symptoms refractory to levodopa, such as freezing of gait. However, it is an important study because this target is not yet well established and is still in the experimental phase.^26^

Among the most cited articles, three validated instruments were identified to assess the quality of life of patients with PD. These instruments include the Parkinson's Disease Questionnaire 39 (PDQ-39),^18,2,28^ the Unified Parkinson's Disease Rating Scale (UPDRS),^19,20^ and the Parkinson's Disease Quality of Life questionnaire (PDQL).^31^ Other studies have associated the PDQ-39 with the Short Form-36 (SF-36)^30^ and the PDQ-39 with the UPDRS.^22^ Finally, one article described the quality of life in a general way.^29^

It is noteworthy that for the evaluation of patients, it is recommended to associate general and specific instruments with evaluating different aspects of quality of life, producing both general data, which facilitate comparisons between different conditions, and related data, specifically on the impact of the disease on quality of life.^37^ However, this association was verified in only one of the most cited studies.^38^

Although not present in Figure 3A, the scientific journal "Lancet Neurology," with the first edition released in October 1823 (Journal Citation Reports™ 2021:59.935), appeared four times in the list of most cited articles. Another scientific journal that gained prominence among publications was "The New England Journal of Medicine," published uninterruptedly for more than 200 years (Journal Citation Reports™ 2021:176.082) and appeared three times in the list of the 15 most cited articles. These findings demonstrate the great scope of these two journals, which are highly relevant to the medical field.

When analyzing the conceptual structure, terms that have already been developed in depth were found to refer to traditional areas related to the study of targets, including the STN, GPi, and basal ganglia. These findings reaffirm the central role that studies on potential targets have played in the subject studied, which has already been addressed when discussing the most cited articles.

The analysis also revealed emerging themes related to DBS in patients with PD, including drug therapy, levodopa, follow-up, and quality of life. This has been gaining prominence as the follow-up of these patients brings up relevant questions about their evolution, such as the combination of drug adjustment with neuromodulation, the importance of considerations about levodopa before and after surgery, and the follow-up of these patients with particular attention to the quality of life, which will ultimately define the success of the treatment.

Through temporal analysis, the trend in the evolution of studies is evident. At the beginning of the research, the study of DBS targets within the thalamocortical basal ganglia loop predominated, with emphasis on the STN, evolving over the years to the approach of cardinal symptoms. Studies have also begun to address axial symptoms (more common in advanced PD), non-motor symptoms, and patients’ quality of life.

Finally, it should be noted that this study has some limitations, considering that only a single database, WoS, was used. Although it is a reference platform for scientific citations intended to support scientific and academic research, it does not cover all the available scientific literature.

## CONCLUSION

In the bibliometric analysis of DBS in treating PD, it was observed that the publication of articles increased until 2020 and has been declining since then. The top scientific Journal was Movement Disorders, with the highest number of publications and citations. Michael Okun and Andrés Lozano have published the most number of articles.

The institutions that concentrated most on authors were the University of Florida and the University of Toronto; however, it was noted that these authors mainly came from countries in the Northern Hemisphere. The most cited articles and conceptual structure demonstrated the focus of studies on the results of surgery, with the GPi and STN described as the primary targets. When considering the temporal analysis, it became evident that recent studies’ addressed axial symptoms (more common in advanced PD), non-motor symptoms, and patients’ quality of life.

Further studies with larger patient cohorts and more randomized controlled trials are required to further elucidate the long-term benefits of this technology on motor symptoms and quality of life.

## References

[1] Weaver FM, Follett KA, Stern M (2012). Randomized trial of deep brain stimulation for Parkinson disease:
thirty-six-month outcomes. Neurology.

[2] Odekerken VJ, Boel JA, Schmand BA (2016). GPi vs STN deep brain stimulation for Parkinson disease:
three-year follow-up. Neurology.

[3] Limousin P, Pollak P, Benazzouz A (1995). Effect of parkinsonian signs and symptoms of bilateral
subthalamic nucleus stimulation. Lancet.

[4] Krack P, Batir A, Van Blercom N (2003). Five-year follow-up of bilateral stimulation of the subthalamic
nucleus in advanced Parkinson's disease. N Engl J Med.

[5] Deuschl G, Antonini A, Costa J (2022). European Academy of Neurology/Movement Disorder Society-European
section guideline on the treatment of Parkinson's disease: I. invasive therapies. Mov Disord.

[6] Limousin P, Foltynie T (2019). Long-term outcomes of deep brain stimulation in Parkinson
disease. Nat Rev Neurol.

[7] Zupic I, Čater T (2015). Bibliometric Methods in Management and
Organization. Organ Res Methods.

[8] Pajo AT, Espiritu AI, Jamora RDG (2020). Scientific impact of movement disorders research output in
Southeast Asian countries: a bibliometric analysis. Parkinsonism Relat Disord.

[9] Listik C, Listik E, Cury RG (2020). Deep brain stimulation treatment in dystonia: a bibliometric
analysis. Arq Neuropsiquiatr.

[10] Yang C, Wang X, Tang X, Wang R, Bao X (2020). Stem-Cell Research of Parkinson Disease: Bibliometric Analysis of
Research Productivity from 1999 to 2018. World Neurosurg.

[11] Hu K, Moses ZB, Xu W, Williams Z (2017). Bibliometric profile of deep brain stimulation. Br J Neurosurg.

[12] Dada O (2018). A model of entrepreneurial autonomy in franchised outlets: A
systematic review of the empirical evidence. Int J Manag Rev.

[13] Rey-Martí A, Ribeiro-Soriano D, Palacios-Marqués D (2016). A bibliometric analysis of social
entrepreneurship. J Bus Res.

[14] Moura LKB, de Mesquita RF, Mobin M (2017). Uses of bibliometric techniques in public health
research. Iran J Public Health.

[15] Sousa AR, Carvalho ARB, Ferreira da Silva MD (2023). Bibliometric analysis of global scientific production on COVID-19
and vaccines. Int J Environ Res Public Health.

[16] Aria M, Cuccurullo C (2017). *bibliometrix*: An R-tool for comprehensive science mapping
analysis. J Informetr.

[17] Araújo CAA (2006). Bibliometria: evolução histórica e questões
atuais. EQ.

[18] Deuschl G, Schade-Brittinger C, Krack P (2006). A randomized trial of deep-brain stimulation for Parkinson's
disease. N Engl J Med.

[19] Obeso JA, Olanow CW, Deep-Brain Stimulation for Parkinson's Disease Study Group (2001). Deep-brain stimulation of the subthalamic nucleus or the pars
interna of the globus pallidus in Parkinson's disease. N Engl J Med.

[20] Weaver FM, Follett K, Stern M (2009). Bilateral deep brain stimulation vs best medical therapy for
patients with advanced Parkinson disease: a randomized controlled
trial. JAMA.

[21] Benabid AL, Chabardes S, Mitrofanis J, Pollak P (2009). Deep brain stimulation of the subthalamic nucleus for the
treatment of Parkinson's disease. Lancet Neurol.

[22] Follett KA, Weaver FM, Stern M (2010). Pallidal versus subthalamic deep-brain stimulation for
Parkinson's disease. N Engl J Med.

[23] Rodriguez-Oroz MC, Obeso JA, Lang AE (2005). Bilateral deep brain stimulation in Parkinson's disease: a
multicentre study with 4 years follow-up. Brain.

[24] Little S, Pogosyan A, Neal S (2013). Adaptive deep brain stimulation in advanced Parkinson
disease. Ann Neurol.

[25] Bronstein JM, Tagliati M, Alterman RL (2011). Deep brain stimulation for Parkinson disease: an expert consensus
and review of key issues. Arch Neurol.

[26] Stefani A, Lozano AM, Peppe A (2007). Bilateral deep brain stimulation of the pedunculopontine and
subthalamic nuclei in severe Parkinson's disease. Brain.

[27] Kumar R, Lozano AM, Kim YJ (1998). Double-blind evaluation of subthalamic nucleus deep brain
stimulation in advanced Parkinson's disease. Neurology.

[28] Williams A, Gill S, Varma T (2010). Deep brain stimulation plus best medical therapy versus best
medical therapy alone for advanced Parkinson's disease (PD SURG trial): a
randomised, open-label trial. Lancet Neurol.

[29] Benabid AL (2003). Deep brain stimulation for Parkinson's disease. Curr Opin Neurobiol.

[30] Witt K, Daniels C, Reiff J (2008). Neuropsychological and psychiatric changes after deep brain
stimulation for Parkinson's disease: a randomised, multicentre
study. Lancet Neurol.

[31] Odekerken VJ, van Laar T, Staal MJ (2013). Subthalamic nucleus versus globus pallidus bilateral deep brain
stimulation for advanced Parkinson's disease (NSTAPS study): a randomised
controlled trial. Lancet Neurol.

[32] Okun MS, Fernandez HH, Wu SS (2009). Cognition and mood in Parkinson's disease in subthalamic nucleus
versus globus pallidus interna deep brain stimulation: the COMPARE
trial. Ann Neurol.

[33] Hubert JJ (1980). Linguistic indicators. Soc Indic Res.

[34] Traag VA, Waltman L, van Eck NJ (2019). From Louvain to Leiden: guaranteeing well-connected
communities. Sci Rep.

[35] Zhong QQ, Zhu F (2022). Trends in Prevalence Cases and Disability-Adjusted Life-Years of
Parkinson's Disease: Findings from the Global Burden of Disease Study
2019. Neuroepidemiology.

[36] Fujikawa J, Morigaki R, Yamamoto N (2022). Therapeutic devices for motor symptoms in Parkinson's disease:
current progress and a systematic review of recent randomized controlled
trials. Front Aging Neurosci.

[37] Tiago MSF, Almeida FO, Santos LS, Veronezi RJB (2010). Instrumentos de avaliação de qualidade de vida na doença de
Parkinson. Rev Neurocienc.

[38] Barcelos RMFM, Sousa GSS, Almeida MV (2021). Qualidade de vida de pacientes com hanseníase: uma revisão de
escopo. Rev Esc Enferm USP.

